# On the Death of Mr. Huskisson

**Published:** 1831-01-01

**Authors:** 


### XXXI.

### MR. HUSKISSON.

DR. WETHERILL of Liverpool, whose uterine operations we some time ago took the liberty to censure, has recently put forth an anathema against the practice pursued by the surgical attendants of the late Mr. Huskisson, strongly stigmatizing them, in the most illiberal language, as pursuing a treatment "unscientific, inefficient, and imbecile." These are terms which the liberal doctor not only uses himself, but puts into the mouths of his brethren generally in Liverpool. We do not believe this last insinuation, not only because the surgical faculty of that large town is better informed on the subject; but because we happen to know their sentiments through a channel on which we can depend. Dr. Wetherill asserts, or copies the assertion, that "an army or a navy surgeon might have saved the life of Mr. Huskisson, and so might any other surgeon, whose head and hands knew how and when to do their duty." The rashness of this assertion, coming from men who were not present at the accident, and who could know nothing of the case beyond what the public papers conveyed, is unbearable! Every surgeon who knows any thing of his profession, is aware that no general rule will apply to all cases of injury requiring amputation. Of ten men, whose thighs may be shattered to pieces by external agents, five may be in a state to bear immediate operation—while the other five may have received such a shock, and have their nervous systems so depressed, that an operation would be madness before some degree of animation was restored. All accounts agree, and Mr. Whatton, in an able reply, published in the Lancet, confirms them, that the lamented Mr. Huskisson was at no period, after the accident, in a state that could at all authorize amputation of the limb, or afford the slightest hope of success from such an operation. We are assured by Mr. Whatton, that a ligature was thrown round the femoral artery immediately, and that there was no hæmorrhage of any consequence. The following short account of the accident and death is worthy of record, and will shew the illiberality of Dr. Wetherill's strictures in a clear point of view.

"Mr. Huskisson received a compound fracture of the leg and thigh, on the 15th of Sept. last; both bones of the leg were broken at the upper third, and much comminuted; their splintered ends exposed, and the soft parts lacerated to a considerable extent; the femur was fractured somewhat above its middle, and both ends exposed; there was here also much comminution, and an extensive laceration of the muscles and integuments, and the femoral vessels were distinctly visible at the bottom of the wound. As the accident happened midway between Manchester and Liverpool, considerable delay took place before one of the carriages could be detached from the train, and the necessary arrangements effected; but as soon as this could be done, Mr. Huskisson was carried forwards to the vicarage at Eccles, and the same engine passed on to Manchester for surgical assistance.

Mr. Ransome, Mr. Garside, Mr. White, and myself, were on the rail-road when the engine arrived; and having learnt the particulars of the accident from Lord Wilton, who came up from Eccles, the necessary instruments for amputation were procured from Manchester, and we set off as quickly as possible for the vicarage, where we arrived at about half-past two o'clock, two hours after the accident.

Mr. Ransome and myself were introduced by Lord Wilton, and found that Dr. Brandreth of Liverpool, and Dr. Hunter of Edinburgh, who had accompanied the procession, had arrived in the carriage with Mr. Huskisson, and had remained in attendance upon him from the period of the injury.

The patient was laid on a sofa; there had been great hæmorrhage, not only on the receipt of the wound, but afterwards, by constant draining from the veins; countenance pale and ghastly, forehead covered with cold perspiration, cold and stiffened extremities, and sickness and oppression at the stomach, with frequent convulsive shudders, difficult respiration, and great constitutional alarm.

Although an immediate amputation was every way desirable, yet, to have operated under these circumstances, would have been madness in the surgeon, and certain death to the patient. Small quantities of warm cordials were given at intervals, as the stomach would bear them, bottles of hot water were applied to the hands, feet, and sides of chest, and every thing was had recourse to, with the view of calming the constitutional disturbance, and of restoring some little power, to enable him to endure the additional shock of the operation.

These efforts were in vain. The anxiety and oppression still remained, the pulse fluttered occasionally at the bend of the arm, the difficulty of breathing increased, the convulsions became gradually more violent, and we witnessed his departure, with feelings of the deepest sympathy and regret, at nine o'clock, 8½ hours after the accident." —Lancet.

We need hardly express our belief, that the medical attendants on this melancholy case are entirely free from blame.

